# Role of Endothelial to Mesenchymal Transition in the Pathogenesis of the Vascular Alterations in Systemic Sclerosis

**DOI:** 10.1155/2013/835948

**Published:** 2013-09-23

**Authors:** Sergio A. Jimenez

**Affiliations:** ^1^Jefferson Institute of Molecular Medicine, Philadelphia, PA 19107, USA; ^2^Scleroderma Center, Thomas Jefferson University, Philadelphia, PA 19107, USA

## Abstract

The pathogenesis of Systemic Sclerosis (SSc) is extremely complex, and despite extensive studies, the exact mechanisms involved are not well understood. Numerous recent studies of early events in SSc pathogenesis have suggested that unknown etiologic factors in a genetically receptive host trigger structural and functional microvascular endothelial cell abnormalities. These alterations result in the attraction, transmigration, and accumulation of immune and inflammatory cells in the perivascular tissues, which in turn induce the phenotypic conversion of endothelial cells and quiescent fibroblasts into activated myofibroblasts, a process known as endothelial to mesenchymal transition or EndoMT. The activated myofibroblasts are the effector cells responsible for the severe and frequently progressive fibrotic process and the fibroproliferative vasculopathy that are the hallmarks of SSc. Thus, according to this hypothesis the endothelial and vascular alterations, which include the phenotypic conversion of endothelial cells into activated myofibroblasts, play a crucial role in the development of the progressive fibrotic process affecting skin and multiple internal organs. The role of endothelial cell and vascular alterations, the potential contribution of endothelial to mesenchymal cell transition in the pathogenesis of the tissue fibrosis, and fibroproliferative vasculopathy in SSc will be reviewed here.

## 1. Introduction

Scleroderma or Systemic Sclerosis (SSc) is an autoimmune disease of unknown etiology characterized by progressive fibrosis of skin and multiple internal organs and prominent and often severe alterations in the microvasculature [1]. Although SSc is the third most common systemic inflammatory autoimmune disease and has the highest case-specific mortality among this group of idiopathic disorders, there is currently no effective disease-modifying therapy for SSc. Therefore, there is an urgent unmet need for the development of effective disease-modifying therapies to improve the devastating health consequences and high mortality caused by the disease. The cells responsible for the severe fibroproliferative process in SSc are activated myofibroblasts, a unique population of mesenchymal cells displaying unique biological functions including increased production of fibrillar type l and type lll collagens, initiation of expression of *α*-smooth muscle actin (*α*-SMA), a molecular marker of activated myofibroblasts, and reduction in the expression of genes encoding extracellular matrix (ECM)-degradative enzymes. The accumulation of myofibroblasts in affected tissues and the persistence of their elevated biosynthetic functions are crucial determinants of the extent and rate of progression of the fibrotic process in SSc, and of its clinical course, response to therapy, prognosis, and overall mortality. The origins of the myofibroblasts responsible for the exaggerated and uncontrolled production of collagen and other ECM proteins in SSc have not been completely elucidated. Extensive research studies have shown that these cells originate from several sources, including expansion of resident tissue fibroblasts and migration and tissue accumulation of bone marrow-derived circulating fibrocytes, or from epithelial cells which have undergone epithelial to mesenchymal transition (EMT). More recent studies, however, have demonstrated that endothelial cells are also capable of undergoing endothelial to mesenchymal transition (EndoMT) and that this transition might be an important source of the mesenchymal cells participating in the fibroproliferative vasculopathy and the fibrotic process in SSc. Thus, this novel mechanism of generation of activated myofibroblasts may represent an important and currently unexplored target for the development of disease-modifying therapeutic interventions for this currently incurable disease.

## 2. SSc Pathogenesis: Overview

**Figure 1:**
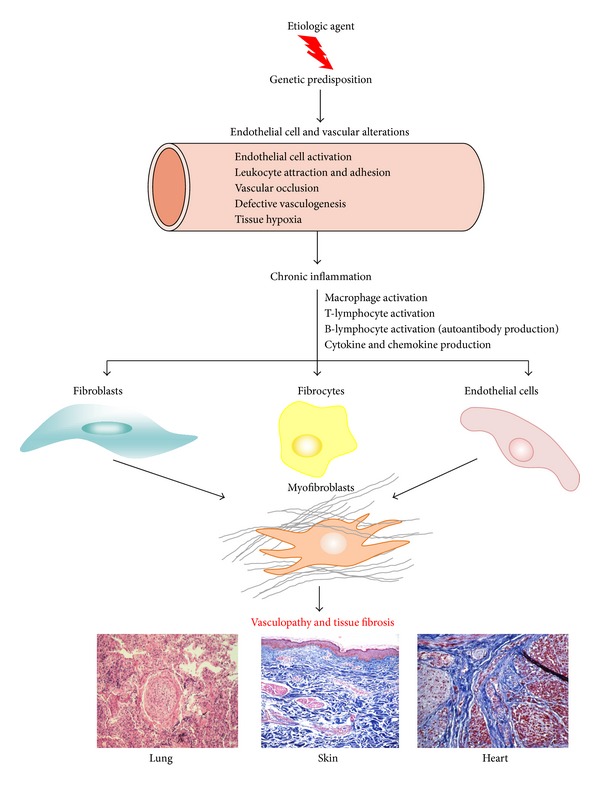
Overall scheme illustrating a current understanding of SSc pathogenesis. Hypothetical sequence of events involved in tissue fibrosis and fibroproliferative vasculopathy in SSc. An unknown causative agent induces activation of immune and inflammatory cells in genetically predisposed hosts resulting in chronic inflammation. Activated inflammatory and immune cells secrete cytokines, chemokines, and growth factors which cause fibroblast activation, differentiation of endothelial and epithelial cells into myofibroblasts, and recruitment of fibrocytes from the bone marrow and the peripheral blood circulation. The activated myofibroblasts produce exaggerated amounts of ECM resulting in tissue fibrosis.

The pathogenesis of SSc is extremely complex, and despite numerous studies that examined several aspects of its intricate picture, the exact mechanisms involved are not well understood. However, it is apparent that the clinical and pathologic manifestations of the disease are the result of three distinct processes: (1) fibroproliferative vascular lesions of small arteries and arterioles, (2) excessive and often progressive deposition of collagen and other ECM macromolecules in skin and various internal organs, and (3) alterations of humoral and cellular immunity characterized by innate immunity alterations, involvement of macrophages and T- and B-lymphocytes, and the production of numerous disease-specific autoantibodies [2–4]. It has not been established which of these processes is of primary importance or how they are temporally related during the development and progression of the disease. However, numerous recent studies have clarified some of the early events in SSc pathogenesis [5–11]. A current hypothesis for SSc pathogenesis posits that there is a sequence of pathogenetic events initiated by unknown etiologic factors in a genetically receptive host which trigger microvascular injury characterized by structural and functional endothelial cell abnormalities. The endothelial cell abnormalities result in the increased production and release of numerous and potent mediators including cytokines, chemokines, polypeptide growth factors, and various other substances such as nitric oxide, prostaglandins, reactive oxygen species (ROS), and thrombogenic and pro-coagulant activities or in the reduction of important compounds such as prostacyclin. The endothelial cell dysfunction triggers the chemokine and cytokine-mediated attraction of specific inflammatory cellular elements from the bloodstream and bone marrow and their transmigration into the surrounding tissues. These events lead to the establishment of a chronic inflammatory process with participation of macrophages and T- and B-lymphocytes, with further production and secretion of cytokines and growth factors that induce the tissue accumulation of activated myofibroblasts, the effector cells responsible for the fibrotic process [12–14]. This sequence of events, diagrammatically illustrated in Figure 1, results in the development of a severe and often progressive fibroproliferative vasculopathy and in the exaggerated and widespread accumulation of fibrotic tissue in the skin and multiple internal organs, which are the most salient characteristics of the disease.

The purpose of this review is to discuss recent studies that have substantially advanced the current understanding of SSc pathogenesis regarding the endothelial cell and vascular abnormalities and the role of endothelial to mesenchymal transition (EndoMT) in the pathogenesis of this currently incurable disease. However, the genetic, innate, and acquired immunological abnormalities and the cytokine, chemokine, and growth factor abnormalities, all of which play an extremely important role in SSc pathogenesis, will not be reviewed here owing to the availability of numerous outstanding reviews about these topics that have been recently published [15–22]. 

## 3. Vascular Abnormalities in SSc

**Figure 2:**
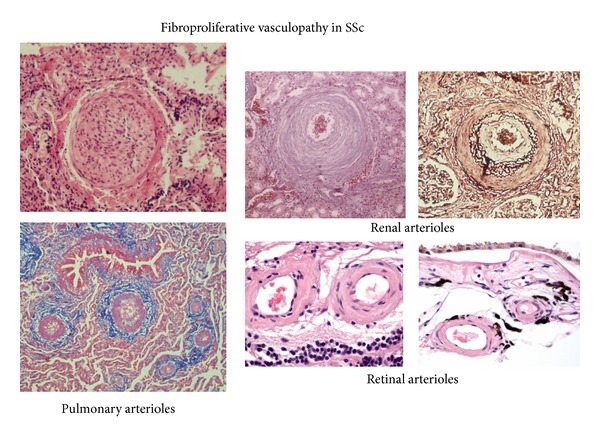
Histopathology of fibroproliferative vasculopathy in small vessels of various affected organs. Histopathology of microvascular arterioles from SSc lung, kidney, and retinal vessels displaying prominent endothelial fibroproliferative alterations causing severe narrowing of vessel lumen and thickening of vessel walls.

Vascular dysfunction is one of the earliest and most noticeable manifestations of SSc as indicated by the occurrence of Raynaud phenomenon, nailfold capillary microvascular alterations, and digital ulcers almost universally in SSc patients often preceding the appearance of clinical evidence of tissue fibrosis [23, 24]. Furthermore, there is a remarkable microvascular fibroproliferative vasculopathy present in essentially all SSc affected organs that is responsible for the most important symptoms and clinical manifestations of SSc and often leads to serious and even fatal complications. Although the effects of vascular abnormalities and dysfunction in patients with SSc are most dramatic when they involve the pulmonary and renal arterioles, causing renal crisis [25, 26] and pulmonary artery hypertension [27–29], respectively, there are numerous other important clinical manifestations of the disease that are caused or mediated by the prominent fibroproliferative vasculopathy. These include capillary rarefaction and capillary loop dilation in the nailfold capillaries [30, 31], cutaneous and mucosal telangiectasias [32–34], erectile dysfunction resulting from alterations in penile blood flow [35–37], and cardiac dysfunction including nonartherosclerotic myocardial infarcts [38, 39], gastric antral vascular ectasia [40–42], central retinal artery occlusion [43, 44], and involvement of larger vessels [45, 46]. Histopathologically, the affected vessels display marked narrowing or even complete occlusion of the vessel lumen with remarkable accumulation of mesenchymal cells and fibrous tissue in the subendothelial compartment and associated endothelial cell abnormalities, which include swelling and apoptotic changes, as well as thickening of the basement membrane. Occasionally, endothelial cell detachment and intravascular platelet thrombi are found. On transmission, electron microscopy universal morphological changes of endothelial cells and basement membrane duplication and lamellation are characteristic alterations. The histopathological changes in the microvasculature of several affected organs are illustrated in Figure 2. 

## 4. Mechanisms of Vascular and Endothelial Cell Injury in SSc

The initial events responsible for the vascular and endothelial cell injury and their subsequent activation are not known although numerous putative etiologic factors have been suggested. Some of these include exogenous chemical substances, vasculotropic viral pathogens, antiendothelial cell antibodies, cellular products from inflammatory cells, tissue hypoxia, or ROS generated during episodes of ischemia/reperfusion [8–10, 47–52]. The injured/activated endothelial cells may undergo apoptosis or may detach from the vascular endothelium, leaving a denuded vascular lumen which triggers the release of endothelial cell precursors from the bone marrow in attempts to repair the endothelial lining defects. Supporting this notion are the observations of increased numbers of circulating endothelial cells and endothelial cell precursors in SSc patients [53–55]. The activation of endothelial cells also induces the expression of cell adhesion molecules such as ICAM, VCAM-1, and E-selectin [56, 57]. The induced expression of cell adhesion molecules by the endothelial cells leads to recruitment and activation of chronic inflammatory cells, including T- and B-lymphocytes and profibrotic macrophage populations and their accumulation in the perivascular tissue and in the interstitium of parenchymal organs. The activated chronic inflammatory cells are responsible for the increased production of transforming growth factor-*β* (TGF-*β*), connective tissue growth factor (CTGF), and other profibrotic polypeptide growth factors which together with the mediators released by the endothelial cells, such as endothelin-1, induce subsequent pathogenetic events leading to the severe tissue fibrosis and fibroproliferative vasculopathy characteristic of the disease [1–11]. Besides the endothelial cell abnormalities, other vascular alterations include increased proliferation of smooth muscle cells in the medial layer of affected vessels, marked accumulation of fibrotic tissue in the subendothelial compartment, and initiation of platelet aggregation and intravascular thrombosis, eventually causing microvascular occlusion [58, 59]. These multiple events result in tissue hypoxia which can cause activation of hypoxia-dependent profibrogenic processes, including further increases in production of TGF-*β* and interstitial collagens as well as other ECM macromolecules [60, 61]. In addition to the structural vascular changes described above, there are also functional vascular alterations which include a reduction in endothelium dependent vasodilator molecules and dysfunction of the neurovascular and neuroendothelial control of vasodilation [62–65], as well as a relative deficiency of vasodilator molecules such as prostacyclin and nitric oxide. 

The injured or cytokine/growth factor-activated endothelial cells also produce increased amounts of the potent profibrotic and vasoconstrictor polypeptide, endothelin-1 [66, 67], and numerous other vasoactive and prothrombogenic compounds that are capable of directly stimulating various target cells such as vascular smooth muscle cells and fibroblasts [8–10, 66, 67]. The important role of endothelin-1 in the development of SSc-associated tissue fibrosis and fibroproliferative vasculopathy has received increasing attention recently. Indeed, elevated levels of endothelin-1 have been found in plasma and bronchoalveolar lavage of SSc patients [68–70] and correlate with clinical parameters and subsets of the disease [71, 72]. Numerous studies have demonstrated that endothelin-1 is a potent inducer of proliferation and ECM production by fibroblastic cells [73–76]. The exaggerated vasoconstrictor response to the increased endothelin levels causes vascular hypoxia and further endothelial injury, thus establishing and maintaining a vicious cycle of endothelial injury and fibrosis. The chronic inflammatory cells accumulated in the perivascular environment also participate in the maintenance of a powerful profibrotic cycle since the numerous cytokines, chemokines, and growth factors they produce can in turn induce further activation of the endothelial cells and their production of profibrotic mediators [67]. The mutual interaction between inflammatory and endothelial cells has been validated by a recent study describing the upregulation of endothelin-1 and TGF-*β* in human microvascular endothelial cells induced by interferon-*γ*, one of the potent cytokines released by the infiltrating inflammatory cells [77]. Additional alterations which contribute to the severe vascular dysfunction and rarefaction in SSc are the result of disordered angiogenesis [78–84] and impaired differentiation of bone marrow stem cells into endothelial cells [85].

## 5. Endothelial to Mesenchymal Transition (EndoMT) in the Pathogenesis of SSc

One of the most characteristic histopathologic alterations in SSc is a severe fibroproliferative vasculopathy affecting the microvasculature as well as some larger vessels [86]. The proliferative vasculopathy of SSc has two distinct components. The first one is a marked proliferation of smooth muscle cells in the media of medium size and small size arterioles, a process which plays a crucial role in SSc-associated pulmonary hypertension. The second component is most prominent in the small arterioles of parenchymal organs, such as the lungs and kidneys, and is characterized by the subendothelial accumulation of activated fibroblasts or myofibroblasts and the production of abundant fibrotic tissue. The origin of mesenchymal cells responsible for the fibrotic microvascular occlusion in SSc is not known, but recent studies have suggested that at least some of these cells may result from EndoMT, that is, the transdifferentiation of endothelial cells into subintimal fibroblasts induced by locally-secreted cytokines and growth factors. During EndoMT, endothelial cells lose their specific endothelial cell markers, such as vascular endothelial cadherin (VE cadherin) and von Willebrand factor, and acquire a mesenchymal or myofibroblastic phenotype initiating expression of *α*-SMA, vimentin, and type I collagen. In addition, these cells become motile and are capable of migrating into surrounding tissues. EndoMT has been described as an important process during cardiac valve and pulmonary artery embryonic development [87–89]. More recently, EndoMT has emerged as a possible mechanism in the pathogenesis of tissue fibrosis in various diseases, including diabetic nephropathy, cardiac fibrosis, intestinal fibrosis, portal hypertension, and pulmonary hypertension [90–100]. Although there is some experimental evidence supporting the participation of EndoMT in SSc, further studies will be required to conclusively demonstrate that EndoMT plays a role in the pathogenesis of SSc-associated fibroproliferative vasculopathy and progressive tissue fibrosis. A firm demonstration of the occurrence of EndoMT in SSc and a further understanding of the molecular mechanisms involved may lead to the pharmacologic modulation or abrogation of this pathway in SSc. 

## 6. Molecular Mechanisms of EndoMT

The molecular mechanisms involved in the EndoMT process have not been fully elucidated, and despite the remarkable importance of this process to normal development and to various pathologic conditions including SSc, only a few studies have examined the molecular changes and the regulatory events occurring in endothelial cells during their transdifferentiation into mesenchymal cells or myofibroblasts. However, substantial recent evidence has accumulated demonstrating the crucial role of TGF-*β* signaling [101–104] in the initiation of EndoMT during normal development as well as in various diseases. 

### 6.1. Role of TGF-*β* in EndoMT

**Figure 3:**
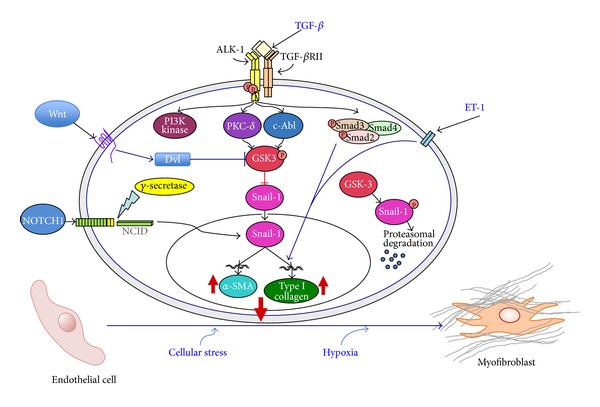
Signaling pathways involved in EndoMT. The diagram shows the numerous putative pathways that may participate in the EndoMT process and may be involved in SSc pathogenesis. One central pathway initiated following ligand-binding activation of the Smad-independent TGF-*β* pathway causes phosphorylation of GSK-3*β* mediated by PKC-*δ* and the c-Abl nonreceptor kinase. Phosphorylation of GSK-3*β* at serine 9 (ser9) causes its inhibition which then allows Snail-1 to enter the nucleus. Nuclear accumulation of Snail-1 results in marked stimulation of Snail-1 expression which then leads to acquisition of the myofibroblast phenotype with stimulation of *α*-SMA. The inhibition of GSK-3*β* ser9 phosphorylation by specific inhibition of PKC-*δ* or c-Abl activity allows GSK-3*β* to phosphorylate Snail-1 targeting it for proteosomal degradation and thus effectively abolishes the acquisition of the myofibroblastic phenotype and the fibrotic response. Other pathways such as those involving ET-1, Wnt, NOTCH, hypoxia, and cellular stress responses may also participate although the molecular events have not been fully elucidated. Modified from Piera-Velazquez and Jimenez [101].

TGF-*β* is a pleiotropic growth factor involved in numerous physiologic and pathologic processes including embryogenesis, cellular development and differentiation, immunologic system development, inflammatory response functions, and wound repair [105–107]. TGF-*β* plays a key role in the pathogenesis of fibrotic diseases by stimulating the production of various collagens and other ECM components by mesenchymal cells and by inhibiting the expression of various relevant metalloproteinases [103, 104, 108–114]. Although the precise mechanisms mediating the potent profibrotic effects of TGF-*β* have not been completely elucidated, it appears that TGF-*β* may cause the establishment of an autocrine signaling cascade capable of continuous activation of profibrotic gene expression in the target cells [115]. However, extensive studies have shown that besides causing a potent stimulation of the expression of genes participating in the exaggerated production and accumulation of ECM, TGF-*β* is also involved in the generation of myofibroblasts through EndoMT [101–104, 116–121]. Indeed, studies in experimentally induced cardiac hypertrophy showed that TGF-*β* was a crucial mediator causing endothelial cells to undergo EndoMT [96]. Although the detailed molecular events and the intracellular cascades activated by TGF-*β* that result in the remarkable phenotypic change of endothelial cells to mesenchymal cells have not been entirely elucidated, recent studies in cultured human cutaneous microvascular endothelial cells [103], primary cultures of murine pulmonary endothelial cells [116], and cultured pancreatic microvascular endothelial cells [121] demonstrated that both Smad-dependent and Smad-independent pathways are involved. The intracellular signaling pathways that are likely to be involved in EndoMT induction by TGF-*β* are illustrated in Figure 3.

Given the crucial role of TGF-*β* in the development of tissue fibrosis and its participation in the pathogenesis of numerous fibrotic diseases, we recently examined the mechanisms involved in the induction of EndoMT by this pleotropic growth factor and studied the intracellular transduction pathways involved in this process employing primary pulmonary endothelial cells in culture [116]. In our study, we examined the transdifferentiation of murine pulmonary endothelial cells into mesenchymal cells *in vitro* and the signaling pathways involved in this process and made the following observations: (1) primary murine pulmonary endothelial cells undergo EndoMT in response to TGF-*β* with initiation of expression of *α*-SMA, assembly of typical intracellular *α*-SMA stress fibers, and loss of VE-cadherin *in vitro*; (2) TGF-*β* induction of EndoMT was associated with a strong upregulation in the expression of the transcriptional repressor Snail-1 indicating that Snail-1 is directly involved in TGF-*β*-induced *α*-SMA expression; and (3) induction of *α*-SMA expression in pulmonary endothelial cells was mediated by the c-Abl kinase and by protein kinase c-*δ* (PKC-*δ*), as specific inhibition of their kinase activity with imatinib mesylate and rottlerin, respectively, or by knockdown of their corresponding transcripts with specific siRNA abrogated the marked increase in TGF-*β* induced *α*-SMA and Snail-1 expression and protein levels. These studies collectively showed that these effects are mediated by the transcriptional repressor Snail-1 [103, 116]. Snail-1 is a zinc-finger transcription factor that forms a complex with Smad3/Smad4. The active Smad3/Smad4/Snail-1 complex causes potent inhibition of the expression of E-cadherin by directly binding to specific sequences within the gene promoter and blocking its transcription. Besides inhibition of E-cadherin, Snail-1 induces numerous transcriptional events that lead to the expression of a mesenchymal-cell-specific phenotype. Snail-1 levels are regulated by complex phosphorylation events mediated by intracellular kinases including c-Abl kinase, PKC-*δ*, PI3K, p38 MAP kinase and glycogen synthase kinase 3*β* (GSK-3*β*). The role of PKC-*δ* and c-Abl kinases has been demonstrated employing specific kinase inhibitors and/or specific knockdown with small interfering RNAs [116], whereas the role of PI3K, p38 MAPK and GSK-3*β* was demonstrated employing specific inhibitors of the corresponding pathways [103]. Numerous studies have shown a crucial role of GSK-3*β* in the regulation of Snail-1 effects. GSK-3*β* phosphorylation results in its inactivation which in turn induces the nuclear accumulation of Snail-1 followed by a profound increase in the expression of its corresponding gene. In contrast, in the absence of GSK-3*β* phosphorylation, the GSK-3*β* kinase is active and induces the proteosomal degradation of Snail-1, thus abrogating the endothelial to mesenchymal cellular phenotypic conversion. The role of GSK-3*β* in the regulation of Snail-1 stability, and therefore, in the expression of its potent transcriptional effects is illustrated in Figure 3.

### 6.2. Regulation of EndoMT by the Wnt and NOTCH Signaling Pathways

Although not extensively studied in EndoMT, it has recently become apparent that several important regulatory pathways including the canonical Wnt pathway and the NOTCH pathway may also participate in the regulation of EndoMT as illustrated in Figure 3. 

#### 6.2.1. Wnt Signaling

The Wnt proteins comprise a large family of secreted glycoproteins with complex canonical and noncanonical intracellular signaling pathways that play crucial roles during embryonic development and organogenesis [122–124]. Wnt proteins and pathways have been recently implicated in the pathogenesis of numerous diseases, including SSc and other fibrotic diseases [125–129]. TGF-*β* appears to be the major factor activating the canonical Wnt pathway [130, 131]. This process is probably mediated by a decrease of Dickkopf-related protein 1 (Dkk-1), a potent Wnt pathway inhibitor, as indicated by the observations that the addition of recombinant Dkk-1 blocked the stimulatory effects of TGF-*β* on the canonical Wnt pathway in fibroblasts [132, 133]. 

Although there is extensive published literature regarding the role of Wnt pathway activation in the phenotypic conversion of epithelial cells into mesenchymal cells, also known as EMT [134–137], the possibility that Wnt may participate in EndoMT is just beginning to be explored. Indeed, a recent study examined the role of Wnt7 and the Wnt7 antagonist Dkk-1 on EndoMT in primary aortic endothelial cells in culture and in transgenic mice with an endothelial-specific Wnt-7b deletion [138]. The results showed that Dkk-1 inhibition of the Wnt pathway enhanced EndoMT, whereas Wnt-7b expression preserved the endothelial cell phenotype. 

#### 6.2.2. NOTCH Signaling

The NOTCH proteins are members of the group of proteins collectively known as morphogens owing to their crucial roles in cell fate decisions during morphogenesis and embryonic development, particularly in relation to cardiovascular development and to regulation of central nervous system polarity and vertebrate segmentation [139, 140]. However, involvement of NOTCH proteins in a broad spectrum of disorders is just becoming apparent [141–147]. The role of NOTCH signaling in EndoMT was first described by Noseda et al. [148], and it was suggested that the NOTCH pathway may be crucial for heart valve and cardiac cushion development and/or vascular smooth muscle differentiation. Numerous subsequent studies have confirmed and extended these observations and have examined the molecular mechanisms involved and the important interactions with the TGF-*β* pathways [149–154]. Studies to examine the participation of NOTCH proteins in the EndoMT process in SSc have not been described, although the demonstration of activation of NOTCH signaling in affected SSc skin suggests that the NOTCH proteins may play a role in SSc pathogenesis and thus may represent a potential target for SSc disease modifying therapeutic approaches [155, 156]. 

### 6.3. Caveolin-1 Regulation of EndoMT

Another recently identified mechanism of regulation and fine tuning of TGF-*β* activity involves Caveolin-1 (Cav-1), the most important member of a family of proteins found in lipid rafts. Cav-1 plays an important role in TGF-*β* signaling regulation owing to its participation in TGF-*β* receptor (T*β*R) internalization [157–159]. T*β*Rs are internalized both by Cav-1-associated lipid rafts and by early endosome antigen 1 (EEA-1) nonlipid raft pathways. Non-lipid raft associated internalization increases TGF-*β* signaling, whereas caveolin-associated internalization increases T*β*R degradation, thereby effectively decreasing or abolishing TGF-*β* signaling [157]. The localization of the T*β*Rs in the EEA-1 positive compartment is responsible for downstream Smad activation, whereas their localization in Cav-1 containing lipid rafts has been shown to cause subsequent receptor ubiquitination and rapid degradation and turnover [158, 159]. Despite the important interactions between Cav-1 and TGF-*β* and the numerous studies that supported the role of Cav-1 in the pathogenesis of SSc [160–164], the possibility that Cav-1 may participate in the regulation of EndoMT has not been explored in detail, although a recent study examined the contribution of Cav-1 to EndoMT employing Cav-1 knockout mice [165]. The results indicated that Cav-1 may be a crucial regulator of EndoMT in murine pulmonary endothelial cells. In these studies, it was shown that pulmonary endothelial cells isolated from Cav-1 knockout mice displayed spontaneous EndoMT and that Cav-1 deficiency potentiated the EndoMT effect induced by TGF-*β* [165].

### 6.4. Role of Other Growth Factors in EndoMT

The most severe clinical and pathologic manifestations of SSc are the result of a fibrotic process characterized by the excessive and often progressive deposition of collagen and other connective tissue macromolecules in skin and numerous internal organs. Numerous studies have shown that tissue fibrosis in SSc is the result of an upregulated expression of genes encoding collagen and other extracellular matrix proteins in affected organs. The exact mechanisms responsible for the establishment of the fibrotic process in SSc have not been precisely determined [2–6], although it has become very clear that several growth factors play a crucial role [166]. 

Besides TGF-*β*, the most important growth factor, involved in SSc tissue fibrosis and in EndoMT, other growth factors and profibrogenic molecules, including platelet derived growth factors [167], vascular endothelial growth factor [168], and insulin-derived growth factor [169], may also participate in EndoMT although their role in this process has not been studied to our knowledge. However, some studies that examined the role of other profibrotic growth factors have been described. One of the profibrotic polypeptides shown to participate in EndoMT is endothelin-1. One study showed that endothelial cell-derived endothelin-1 promotes cardiac fibrosis and heart failure in diabetic hearts through stimulation of EndoMT as these effects did not occur in hearts from transgenic mice with endothelial cell specific endothelin-1 deletion [170]. 

Connective Tissue Growth Factor (CTGF), also known as CCN2, is another pleotropic growth factor that has emerged as an important mediator of normal and pathological tissue fibrotic responses [171–174] and has been suggested to play a crucial role in SSc tissue fibrosis. TGF-*β* causes potent stimulation of CTGF synthesis in fibroblasts, vascular smooth muscle cells, and endothelial cells, and numerous studies have shown that CTGF represents a downstream mediator of TGF-*β* fibrogenic effects [175–177]. Despite the important role of CTGF in the pathogenesis of tissue fibrosis and its potential participation in SSc owing to the well-recognized functional interactions with TGF-*β*, its possible participation in the EndoMT process has not been investigated, although a very recent study showed that elevated levels of CTGF in SSc microvascular endothelial cells were capable of stimulating fibroblast activation and increased motility and invasion in *in vitro* studies. Further investigation indicated that these effects were mediated by CTGF-induced increased expression of TGF-*β* in the target fibroblasts [178]. Another very recent study demonstrated that CTGF is one of the target genes of Snail-1 and showed a remarkable increase in CTGF expression in endothelial cells following experimentally-induced overexpression of Snail-1 [179].

### 6.5. Role of MicroRNAs in the Regulation of EndoMT

MicroRNAs (miRNAs) are small (~22 nucleotides), evolutionarily conserved noncoding RNAs which play important roles in the regulation of the expression of a large number of protein coding genes at the posttranscriptional level [180–183]. The mechanisms involved in posttranscriptional regulation of gene expression by miRNAs are complex and require the sequence-specific complementary binding to the 3′ untranslated region (UTR) of target mRNAs suppressing their expression by either inhibiting mRNA translation or facilitating their degradation [184–186]. Recent interest has been devoted to elucidating their participation in tissue fibrosis and fibrotic diseases [187–190]. Indeed, several miRNAs have been shown to be involved in SSc tissue fibrosis [191–197], displaying either profibrotic or antifibrotic effects. Furthermore, it has been shown that numerous miRNAs display strong modulation of their expression by TGF-*β* [198], although the implications of these TGF-*β*-miRNA interactions have not been fully elucidated. Moreover, several studies have described potential modulatory effects of miRNA on EMT [199], although their participation in EndoMT has not been examined in detail. However, recent reports described results indicating that miRNA21 partially mediated the TGF-*β*-induced EndoMT in human umbilical vein endothelial cells [200] and that several miRNAs were either increased or decreased during TGF-*β*2-induced EndoMT in murine cardiac endothelial cells [120]. 

## 7. Conclusions and Future Directions

Scleroderma or Systemic Sclerosis (SSc) is a systemic autoimmune disease of unknown etiology characterized by progressive fibrosis of skin and multiple internal organs and severe alterations in the microvasculature [1]. SSc is the third most common systemic inflammatory autoimmune disease and has the highest case-specific mortality among this group of idiopathic disorders. Whereas remarkable therapeutic advances have recently been accomplished for Rheumatoid Arthritis and Systemic Lupus Erythematosus, there is currently no effective disease-modifying therapy for SSc. Therefore, there is an urgent unmet need for the development of effective disease-modifying therapies to improve the devastating health consequences and high mortality caused by the disease. The effector cells ultimately responsible for the severe fibroproliferative process in SSc are activated myofibroblasts. These cells display very active protein synthesis producing increased amounts of ECM proteins and acquiring a motile and contractile phenotype expressing a high level of *α*-SMA [12–14]. Although it is widely recognized that there are numerous inflammatory and immunological events in the pathogenesis of SSc, myofibroblasts have been recognized as the crucial determinant of the fibrotic process in SSc and other fibrotic disorders [6, 11–13, 201–203]. Furthermore, their accumulation in affected tissues and the persistence of their elevated biosynthetic functions are the primary determinants of the extent and severity of the clinical manifestations in SSc, and of its clinical course, response to therapy, prognosis, and overall mortality. Thus, activated myofibroblasts have become an important target for SSc disease-modifying therapeutic approaches [204–206]. 

Extensive research studies have shown that these cells originate from several sources [94, 207], including expansion and phenotypic activation of resident tissue fibroblasts and migration and tissue accumulation of bone marrow-derived circulating fibrocytes, or from epithelial cells which have undergone EMT. More recent studies, however, have demonstrated that endothelial cells are also capable of undergoing a phenotypic change to activated mesenchymal cells in a complex process known as EndoMT. Although there are very few studies that have examined the possible participation of EndoMT in the initiation and progression of the fibrotic and fibroproliferative processes in SSc, it is expected that given its potential importance in the pathogenesis of this currently incurable disease this area of investigation may attract further scientific attention. 

Despite the relatively recent research interest in the role of EndoMT in the SSc pathogenesis, important components of the complex pathway of TGF-*β*-induced EndoMT and the molecular mechanisms involved in the generation of activated tissue myofibroblasts have already been identified. These observations suggest that targeting components of these pathways may be a feasible therapeutic goal to modify crucial steps in the development of SSc fibroproliferative vasculopathy [208]. Furthermore, the important role that miRNAs have been shown to play in the regulation of gene expression has clearly opened the possibility of developing a novel therapeutic approach for SSc by targeting these extremely versatile noncoding RNA species. Obviously, subsequent preclinical studies employing suitable animal models will be required to further support the potential therapeutic role of EndoMT and/or miRNA modulation for the fibrosis and proliferative vasculopathy of SSc.
